# Tit for tat? A study on the relationship between work connectivity behavior after-hours and employees’ time banditry behavior

**DOI:** 10.3389/fpsyg.2023.1322313

**Published:** 2024-04-26

**Authors:** Jingya Li, Hao Chen, Liang Wang, Jiaying Bao

**Affiliations:** ^1^School of Economics and Management, Ningxia University, Yinchuan, Ningxia, China; ^2^School of Public Health and Management, Youjiang Medical University for Nationalities, Baise, Guangxi, China; ^3^School of Economics and Management, Wuxi Vocational Institute of Arts and Technology, Wuxi, Jiangsu, China; ^4^School of Languages and Cultures, Youjiang Medical University for Nationalities, Baise, Guangxi, China

**Keywords:** work connectivity behavior after-hours, work alienation, psychological distress, employees’ time banditry behavior, organization-based self-esteem

## Abstract

Based on Conservation of Resources Theory, this study tries to reveal the mechanism of action of work connectivity behavior after-hours triggering employees’ time banditry behavior. By using Mplus7.4 software the analysis of 429 leader-employee paired data collected in three stages reveals that work connectivity behavior after-hours has a positive effect on work alienation and psychological distress. Work alienation and psychological distress mediates the relationship between work connectivity behavior after-hours and employees’ time banditry behavior, respectively. In addition, organization-based self-esteem mitigates the positive effects of work connectivity behavior after-hours on work alienation and psychological distress, which in turn also moderates the indirect effects of work connectivity behavior after-hours on employees’ time banditry behavior through work alienation and psychological distress, respectively. This study provides practical guidance for organizations to reduce employee time banditry behavior and human resource management practices in the new technological environment.

## Introduction

With the rapid development of information technology and the dramatic change in the way work interacts, mobile devices and various social media are ubiquitous in the organization’s internal and external work, allowing employees in traditional office environments to stay connected to their work at all time even after they leave the office (Sarros et al., 2002). However, while the use of mobile communication devices has lifted the time and space constraints of work (Pauli et al., 2023), it has also had an impact on the non-work domain of employees, such as the use of mobile devices for work-related activities in the non-workplace or during non-working hours, i.e., “work connectivity behavior after-hours” (Richardson and Benbunan-Fich, 2011).

Although the work flexibility and autonomy brought by the work connectivity behavior after- hours helps employees to better control their work schedule. However, due to environmental and individual variability, the overuse of this work pattern in organizations has gradually revealed its drawbacks, such as increasing the intensity of employees’ work during non-working hours (Dettmers et al., 2016), damaging employees’ physical and mental health (Dettmers et al., 2016), elicits negative emotional reactions from employees (Butts et al., 2015), reduces work engagement (Ter Hoeven et al., 2016), and affects employees’ work attitudes and job performance (Sonnentag and Niessen, 2020).

Previously, research on the impact of work connectivity behavior after-hours in the work field mainly focused on exploring its effects on employees’ work attitudes, in-role work behaviors, and job performance, while the impact of employees’ out-of-role behavior needs to be further explored. Time banditry behavior refers to employees who engage in unauthorized activities unrelated to work during working hours (Bennett and Robinson, 2000; Martin et al., 2010). Studies show that work pressure is an important cause for triggering employees’ time encroachment behavior (Brock, 2010). As an extension of work demands to non-work areas (Gadeyne et al., 2018), work connectivity behavior after-hours can put employees in a stressful “on-call” situation that consumes their physical and mental resources. In order to release this pressure, employees are likely to relax and replenish their individual resources through time banditry behavior. Based on the analysis above, it is worth exploring whether and how work connectivity behavior after-hours affects employees’ time banditry behavior.

Conservation of Resources Theory suggests that when individuals suffer from resource depletion, face the threat of resource depletion, or are in a situation where they invest a lot of resources but lack the resources to replenish them, they can have psychological pressure and they try to preserve existing resources and find new ones (Hobfoll, 1989). Work alienation is a state of mind that arises when individuals perceive that their work does not meet their needs and expectations, and is a negative experience of psychological separation and behavioral alienation from work and the workplace (Li et al., 2023). Psychological distress is a psychological state including depression, stress, anxiety, etc. It is a psychological problem that occurs when an individual is stressed, emotionally depleted, depressed at work (Sun et al., 2023). Related studies have confirmed that work stress from work connectivity behavior after-hours depletes employees’ positive emotions and other physical and mental resources (Garrick et al., 2018), and in order to acquire new resources, employees exhibit more time banditry behaviors (Ding et al., 2018). Therefore, it is likely that work alienation and psychological distress play a mediating role in the relationship between work connectivity behavior after-hours and employees’ time banditry behavior, respectively.

In addition, based on Conservation of Resources Theory, when employees are in a state where emotional resources and other physical and mental resources are excessively depleted, they will replenish individual resources through time banditry behavior and other means. Organization-based self-esteem, as a positive psychological resource (Sujatha et al., 2023), may reduce the possibility of employees with work alienation and psychological distress to acquire new resources through time banditry behavior.

In summary, in this study, the influence mechanism of work connectivity behavior after-hours will be explored from the perspective of “resource gain and loss” by taking two psychological states of employees caused by work connectivity behavior after-hours (alienation from work) and themselves (psychological distress) as an opportunity. At the same time, through the employee’s own subjective cognition (organization-based self-esteem), this study intends to explore whether the two mental states will bring gains in resources, providing valuable references for organizations to correctly view work connectivity behavior after-hours and reduce employees’ time banditry behavior in practical management.

## Literature review and hypotheses

### Work connectivity behavior after-hours is associated with work alienation and psychological distress

Work connectivity behavior after-hours refers to the behavior of employees using various types of portable communication devices (e.g., laptops or handheld devices, etc.) to participate in work or contact colleagues for work purposes during off-hours (Richardson and Benbunan-Fich, 2011), which is manifested by the fact that employees can use cell phones, the Internet, E-mail, QQ, or We-chat to keep in touch with work-related people at work, affecting employees’ positive emotional attitudes and triggering negative consequences such as reduced work well-being and emotional exhaustion (Garrick et al., 2018).

Work alienation refers to a psychological state in which employees are separated from their jobs because their jobs do not meet their needs or expectations (Banai et al., 2004), emphasizing a series of subjective, negative psychological experiences of the work environment, work content, etc., (Nair and Vohra, 2009; Li et al., 2023). Studies have shown that work characteristics are the main causes of work alienation among employees, such as work autonomy, work stress, and interpersonal relationships (He and Zhou, 2023).

Conservation of Resources Theory suggests that people are motivated to acquire, conserve and maintain resources, and the potential or real loss of resources is a threat to them (Hobfoll, 1989, 2001) and that individuals’ resource protection mechanisms will be activated when their own resources are threatened. However, this protective mechanism also depletes individual resources and thus a spiral of resource loss may occur, ultimately leading to a more rapid depletion of resources for managing emotions and cognition (Bacharach and Bamberger, 2007). Negative psychological states emerge when individuals lack sufficient resources for effective emotional and cognitive management. This study argues that work connectivity behavior after-hours may trigger the emergence of employee work alienation.

Specifically, work connectivity behavior after-hours may make it difficult for employees to detach from work both mentally and physically after work, and the extra work pressure may cause some employees to feel threatened by work in their lives and family spheres. This “hidden overtime” will continue to drain employees’ physical energy and effort, thus making them feel the loss of personal resources. The loss of personal resources can reduce employees’ emotional and cognitive self-control (Christian and Ellis, 2011), and the resulting stress and threat may trigger feelings of work alienation. At the same time, employees feel strongly that they are not dominating and controlling their work, but are dominated and controlled by their work, and feel a deep emotional state of helplessness and emptiness, which ultimately leads to an increased sense of work alienation.

In addition, because of the work connectivity behavior after-hours, employees bring their work to the non-working area, which takes away resources (time and energy) that should be used in their life and family area, and makes them lack resources to devote to their life and family area to meet their family belonging needs, which prevents them from enjoying their own life or spending time with their family during non-working time, and leads to employees’ feeling of alienation due to the inability of the work situation to meet their needs. Based on the above discussion, we propose the following hypothesis.

*H1:* Work connectivity behavior after-hours has a positive effect on work alienation.

Psychological distress, as a negative psychological state, generally refers to employees’ psychological problems such as work tension, emotional exhaustion, depression and anxiety at work, which have serious negative effects on their physical and mental health, work attitude and performance (Sun et al., 2023). This study holds that work connectivity behavior after-hours may lead to psychological distress among employees. According to Conservation of Resources Theory, work connectivity behavior after-hours results in greater work responsibilities, additional work tasks, more and greater work challenges, which cause employees to perceive a continuous depletion and threat to the valuable resources they need.

In addition, resource depletion and threat are key factors in causing negative psychological states (Hobfoll, 1989). When they found that resources are greatly deprived and it is difficult to cope with numerous work requirements, individuals exhibit anxiety (Schmidt et al., 2014), emotional exhaustion (Maslach et al., 2001), cognitive stress reactions (Albertsen et al., 2010), etc. As a painful work experience, work connectivity behavior after-hours can cause employees to feel deprived of resources and then fall into a low-pressure emotional vortex from which they cannot escape, resulting in a state of psychological distress. At the same time, employees’ failure to obtain expectations and rewards that match their better and stronger selves due to greater work responsibilities, more tasks, and greater work challenges may also exacerbate their level of psychological distress. Therefore, we propose the following hypothesis.

*H2:* Work connectivity behavior after-hours has a positive effect on psychological distress.

### The mediating role of work alienation and psychological distress

As a productive deviant behavior that is non-invasive and directed towards the organization (Martin et al., 2010), time banditry behavior is a specific type of employee theft against organizational time resources, which is mainly induced by employees’ negative work attitudes, excessive work stress, perceived unfairness, work boredom or work idleness (Brock et al., 2013. Ketchen et al., 2008; Martin et al., 2010). Related studies point out that the negative effects of work connectivity behavior after-hours also permeate the work domain and have a significant impact on employees’ work attitudes and behaviors (Schlachter et al., 2018; Van Laethem and Van Vianen, 2018).

According to Conservation of Resources Theory, when individuals perceive or actually experience a loss of resources at work, they adopt specific behaviors to defend or supplement them in order to compensate for the lost resources and restore resource balance (Hobfoll, 1989). The present study argues that work connectivity behavior after-hours positively influences their time banditry behavior through the mediating effect of work alienation. Specifically, work connectivity behavior after-hours occurs outside of the employee’s schedule, forcing the employee to put aside current activities to respond to work messages in a timely manner, which may interfere with the recovery and replenishment of individual resources and predispose the employee to feelings of work detachment.

Work alienation as a psychological state can have an important impact on employees’ behavior, and passive resistance is the most common response of employees when facing work alienation (Hodson and Sullivan, 2012). Employees with work alienation have lower job satisfaction and motivation, tend to engage in destructive behaviors (e.g., deliberate sabotage, etc.) to restore perceptions of control and internal balance (Ambrose et al., 2002), they may restore resource conservation and alleviate negative psychological states by performing time banditry behaviors (Spector and Fox, 2002. Krischer et al., 2010), helping them release the negative emotions brought about by feelings of work alienation. In summary, work connectivity behavior after-hours can lead to a loss of individual resources, which can reduce employees’ emotional and cognitive self-control and create psychological stress, resulting in feelings of work detachment, which in turn leads to the implementation of time banditry behavior. Therefore, we propose the following hypothesis.

*H3:* Work alienation mediates the relationship between work connectivity behavior after-hours and employees’ time banditry behavior.

Based on Conservation of Resources Theory, this study argues that work connectivity behavior after-hours may lead to psychological distress, and then induce time banditry behavior in employees. Specifically, work connectivity behavior after-hours prevents employees from disengaging from work (Jarvenpaa and Lang, 2005), and the accompanying work overload also imposes psychological stress on employees (Ter Hoeven et al., 2016), and generates negative emotions such as restlessness, tension, and fatigue, resulting in a state of psychological distress. As a kind of workplace deviant behavior that is not easily perceived by others, employees’ time banditry behavior can relieve employees’ negative emotions, relieve work stress and work fatigue, and is easily seen as an effective way to replenish energy by employees who are stressed at work, dissatisfied with their jobs or hold strong perceptions of unfairness and work frustration (Martin et al., 2010).

Related studies have also shown that stress caused by a lack of work resources is one of the important factors influencing employees’ time banditry behavior (Coker, 2011). In summary, work connectivity behavior after-hours overstretches individual resources and causes employees to feel “low energy” during working hours. Therefore, they are likely to release stress and relieve fatigue by encroaching on organizational time resources such as slacking. Thus, we propose the following hypothesis.

*H4*: Psychological distress mediates the relationship between work connectivity behavior after-hours and employees’ time banditry behavior.

### The moderating role of organization-based self-esteem

Organization-based self-esteem is an employee’s self-perception and judgment of their importance in the organization, i.e., the individual’s perception of his or her usefulness to the organization and their ability to create value for the organization (Pierce et al., 1989). Organization-based self-esteem, as the perceived self-worth and importance of organizational members, depends on how individuals feel in organizational situations. Therefore, employees with high organization-based self-esteem feel that they are important, influential, and valuable in the organization (Jahanzeb and Bouckenooghe, 2023) and that their needs are met through role fulfillment in the organization (Pierce and Garden, 2004), whereas employees with low organization-based self-esteem perceive themselves as unimportant and worthless in the organizations they work in. Research has shown that employees with high organization-based self-esteem have more positive self-evaluation and subjective efficacy and produce more constructive behaviors (Chen and Aryee, 2007).

This study argues that organization-based self-esteem can mitigate the relationship between work connectivity behavior after-hours and work alienation. According to Conservation of Resources Theory, individuals have limited resources, and organization-based self-esteem, as a subjective perception, can be viewed as an important personal resource for employees (Pierce and Garden, 2004). Specifically, the higher the level of organizational self-esteem is, the stronger the employee’s perception that the self plays an indispensable role in the organization is, the more mentally and psychologically fulfilled they are, the more pleasurable experiences they feel, and thus the higher level of positive emotions displays, and the better to supplement the available resources. That is, employees with higher organization-based self-esteem are also more willing to accept and less likely to feel detached from work when work connectivity behavior after-hours occurs.

On the contrary, employees with a low organization-based self-esteem lack confidence in themselves and have difficulty in having their competence and role needs met in the organization, making it difficult to maintain a positive emotional state at work and exhibit lower positive emotions, which is extremely likely to result in additional resource loss. As a result, employees with low organization-based self-esteem are full of complaints and dissatisfaction with the organization, and even resist the occurrence of work connectivity behavior after-hours, which in turn increases the sense of work alienation. Thus, we propose the following hypothesis.

*H5:* Organization-based self-esteem moderates the relationship between work connectivity behavior after-hours and work alienation, that is, the higher the organizational self-esteem is, the weaker the positive relationship between work connectivity behavior after-hours and work alienation is.

In addition, this study also holds that the strength of the relationship between work connectivity behavior after-hours and psychological distress is affected by organization-based self-esteem. Specifically, employees with high organization-based self-esteem have strong self-confidence, show positive emotional responses at work, believe that they can be competent for their role in the organization, and believe that their behavior can be beneficial to the organization. However, employees with low organization-based self-esteem are more sensitive to negative organizational situations, and tend to have negative work attitudes and behaviors, thus exacerbates psychological distress. Thus, we propose the following hypothesis.

*H6:* Organization-based self-esteem moderates the relationship between work connectivity behavior after-hours and psychological distress, that is, the higher the organization-based self-esteem is, the weaker positive relationship between work connectivity behavior after-hours and psychological distress is.

### The moderated mediating effects

Based on Conservation of Resources Theory and combined with the above hypotheses, it can be further inferred that the mediating effects of work alienation and psychological distress may be influenced by organization-based self-esteem, i.e., work connectivity behavior after-hours may trigger employees’ work alienation and bring about a state of psychological distress, leading employees to use time banditry behavior as a way of confrontation to obtain psychological balance. However, organization-based self-esteem gives as a supplement to individual resources, which not only reduces the negative effects of connectivity behaviors on work alienation and psychological distress, but also reduces the indirect effects of work connectivity behavior after-hours on time banditry behavior through work alienation and psychological distress, respectively.

Specifically, when employees’ organization-based self-esteem is high, employees are more willing to accept work connectivity behavior after-hours, inhibit work detachment and psychological distress, and are less likely to engage in time banditry behavior. When employees’ organization-based self-esteem is low, the impact of work connectivity behavior after-hours on employees’ work alienation and psychological distress is greater, and employees are more likely to engage in time banditry behavior. Thus, we propose the following hypothesis.

*H7:* Organization-based self-esteem moderates the mediating effect of work alienation on work connectivity behavior after-hours and employees’ time banditry behavior, that is, the higher the organization-based self-esteem is, the weaker the mediating effect between work alienation on work connectivity behavior after-hours and employees’ time banditry behavior is.

*H8:* Organization-based self-esteem moderates the mediating effect of psychological distress on work connectivity behavior after-hours and employees’ time banditry behavior, that is, the higher the organization-based self-esteem is, the weaker the mediating effect between psychological distress on work connectivity behavior after-hours and employees’ time banditry behavior is.

**Figure 1:**
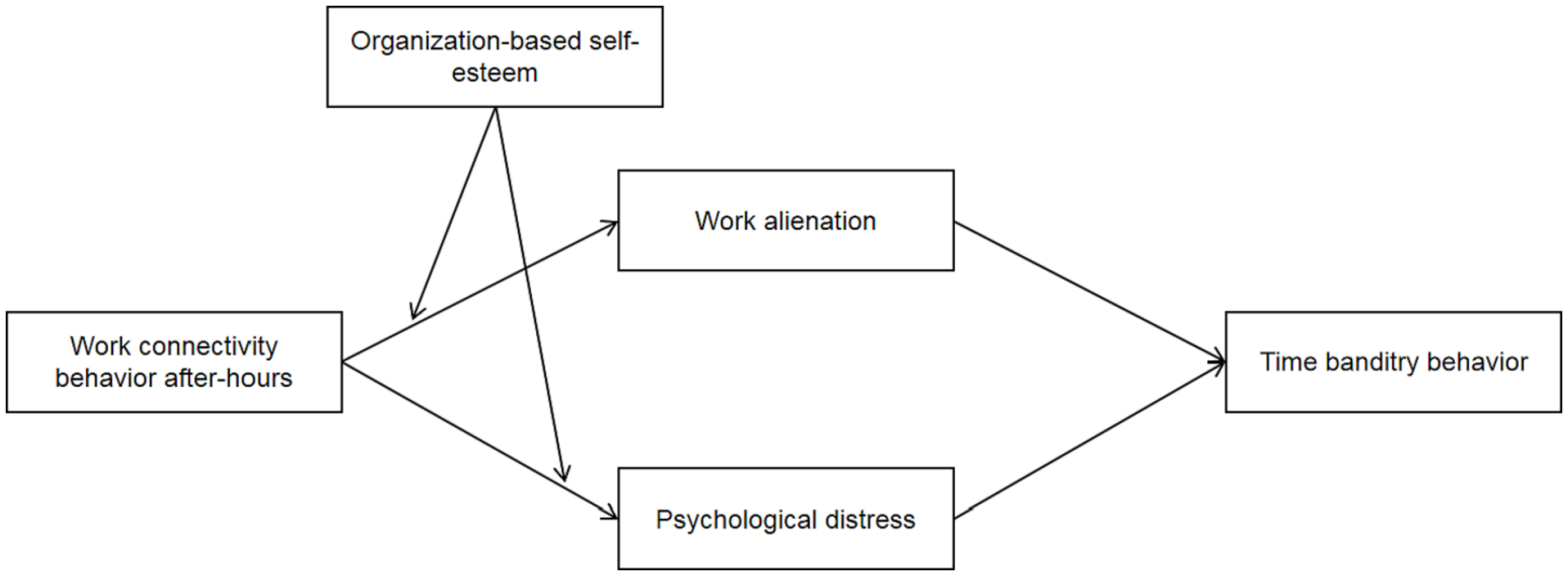
The theoretical model.

The theoretical model is shown in Figure 1.

## Methods

### Participants and procedure

We collected our data through offline questionnaires from existing employees and their direct supervisors of five service-oriented companies in Henan Province China engaged in security, escort, security inspection, and prevention of security technologies. In order to mitigate the effects of homologous bias, this study conducted a 1:1 employee-direct supervisor matching approach to data collection at three-time points, with an interval of 1 month. The survey process was as follows: at time point 1 (T1), survey was for employees, which included basic information about the employee and work connectivity behavior after-hours; at time point 2 (T2), the survey was administered to the employees, which included work alienation, psychological distress and organizational based self-esteem; at time point 3 (T3) the survey was administered to the employees’ direct supervisors, which included employees’ time banditry behavior. Except for some demographic variables, a Likert 6-point scale was used to assess all of the surveys in this study.

In order to enable participants to complete the questionnaire correctly and effectively, we obtained a roster of research subjects with the cooperation of the human resources departments of relevant enterprises, and coded the research subjects based on the organizational structure of each department from the roster. Before the start of the research, we also shared with the participants the purpose of this study and ensured that the results of this survey would be strictly confidential and only used for academic research, in order to alleviate the concerns of participants and improve the accuracy of measurements.

In the first survey, 485 employee questionnaires were distributed on-site, and 472 valid questionnaires were returned. In the second survey, targeted distribution was made to employees who provided valid questionnaires in the first survey and a total of 446 valid questionnaires were returned. In the third survey, targeted questionnaires were distributed to the direct supervisors of employees who provided valid questionnaires for the second time, and finally 429 valid matched questionnaires between employees and direct supervisors were obtained, with a return rate of 88.45%. In terms of sample structure, male employees predominate, accounting for 64% of the total sample. In terms of age structure, young people predominate, with 82.1% of employees under the age of 35. In terms of education structure, bachelor’s degree and above account for 74.7% of the total sample size.

### Measurements

The measurement scales used in this study are mature or widely used by scholars both at home and abroad. Each item was scored on a Likert 6-point scale, and SPSS 24.0 was used to measure the reliability of five main variables of work connectivity behavior after-hours, work alienation, psychological distress, employees’ time banditry behavior and organization-based self-esteem.

For the measurement of work connectivity behavior after-hours, the questionnaire is divided into two dimensions including duration and frequency of occurrence. Richardson and Thompson (2012) suggest that measuring work connectivity behavior after-hours requires the measurement of both frequency and duration dimensions. The duration component of this paper adopts a 4-question scale developed by Richardson and Thompson (2012), e.g., “The length of time I spend on work-related tasks on weekend vacations,” with scales set to “1–15 min,” “16–30 min,” “31–60 min,” “1–2 h” and “more than 2 h.” The frequency component of the scale is adapted from Boswell and Olson-Buchanan's (2007) 4-question scale on work-related communication tools, such as “How often I handle work-related matters by e-mail during non-working hours,” the Cronbach’s α is 0.875.

For the measurement of work alienation, this study adopts the scale developed by Nair and Vohra (2009), which has four items in total. Representative item is “Facing my daily work tasks, I feel painful and bored,” the Cronbach’s α is 0.863.

For the measurement of psychological distress, this study adopts the scale developed by Kessler and Mroczek (1994), which has ten items in total. Representative item is “I am full of energy,” the Cronbach’s α is 0.889.

For the measurement of employees’ time banditry behavior, this study adopts the scale developed by Bennett and Robinson (2000), which has three items in total. Representative item is “I take longer breaks at work than allowed,” the Cronbach’s α is 0.858.

For the measurement of organization-based self-esteem, this study adopts the scale developed by Pierce et al. (1989), which has ten items in total. Representative item is “I am valued in the organization,” the Cronbach’s α is 0.882.

For the measurement of control variables, with reference to previous studies (Chen et al., 2022; Wei et al., 2023), this study selects employees’ age, gender, and education level from common demographic variables as control variables.

## Results

### Confirmatory factor analysis

In this research, confirmatory factor analysis was performed using Mplus 7.4 on relevant variables to test the discriminant validity between variables. The results are shown in Table 1, and the five factor model has the best fitting effect (*χ*^2^ = 222.794, *df* = 125, *χ*^2^*/df* = 1.782, CFI = 0.972, TLI = 0.965, RMSEA = 0.043, SRMR = 0.039), which meets the standards recognized by the academic community and is significantly better than other models (Schermelleh-Engel et al., 2003), indicating good discriminant validity among the five variables in this study. Meanwhile, the single factor model has the worst fitting effect (*χ*^2^ = 1906.161, *df* = 135, *χ*^2^/*df* = 14.120, CFI = 0.486, TLI = 0.417, RMSEA = 0.175, SRMR = 0.136), to some extent, confirming that the data in this study does not have a serious issue of homologous bias, and can be further tested for the relationship between variables.

**Table 1:** Confirmatory factor analysis results of measurement models.

Model	Factor	*χ* ^2^	*df*	*χ*^2^/*df*	CFI	TLI	RMSEA	SRMR
Model1	WCBA + WA + PD + TBB + OBSE	1906.161	135	14.120	0.486	0.417	0.175	0.136
Model2	WCBA + WA + PD + TBB, OBSE	854.038	134	6.373	0.791	0.761	0.112	0.090
Model3	WCBA + WA + PD, TBB, OBSE	737.814	132	5.590	0.824	0.796	0.103	0.081
Model4	WCBA + WA, PD, TBB, OBSE	433.898	129	3.364	0.911	0.895	0.074	0.057
Model5	WCBA, WA, PD, TBB, OBSE	222.794	125	1.782	0.972	0.965	0.043	0.039

*N* = 429; WCBA, work connectivity behavior after-hours; WA, work alienation; PD, psychological distress; TBB, time banditry behavior; OBSE, organization-based self-esteem; +: Two factors combined as one.

### Correlation analysis

This study adopted SPSS 24.0 to measure the mean, standard deviation, and correlation coefficient matrix of each variable. According to Table 2, it can be seen that the correlations between the variables are consistent with the previous hypotheses of this study. Work connectivity behavior after hour is significantly positively correlated with work alienation (*γ* = 0.379, *p* < 0.01), psychological distress (*γ* = 0.424, *p* < 0.01), and employees’ time banditry behavior (*γ* = 0.235, *p* < 0.01). Work alienation is significantly positively correlated with employees’ time banditry behavior (*γ* = 0.296, *p* < 0.01). Psychological distress is significantly positively correlated with employees’ time banditry behavior (*γ* = 0.288, *p* < 0.01).

**Table 2:** Means, standard deviations and correlations of study variables.

	1	2	3	4	5	6	7	8
1.Age								
2.Gender	0.059							
3.Education	0.023	−0.035						
4.Work connectivity behavior after-hours	0.047	0.108^*^	−0.124^*^	(0.875)				
5.Work alienation	0.097^*^	0.108^*^	−0.017	0.379^**^	(0/863)			
6.Psychological distress	0.050	0.096^*^	−0.160^**^	0.424^**^	0.011	(0/889)		
7.Time banditry behavior	0.050	0.045	−0.218^**^	0.235^**^	0.296^**^	0.288^**^	(0.858)	
8.Organization-based self-esteem	0.186^**^	0.025	−0.125^**^	−0.052	−0.014	0.071	−0.116^**^	(0.882)
Mean (M)	31.57	0.28	2.21	4.878	5.047	4.955	5.113	2.535
Standard deviation (SD)	6.848	0.452	0.536	0.715	0.716	0.745	0.671	1.330

*N* = 429; ^*^p < 0.05; ^**^p < 0.01; ^***^p < 0.001.

### Main effects testing

**Figure 2:**
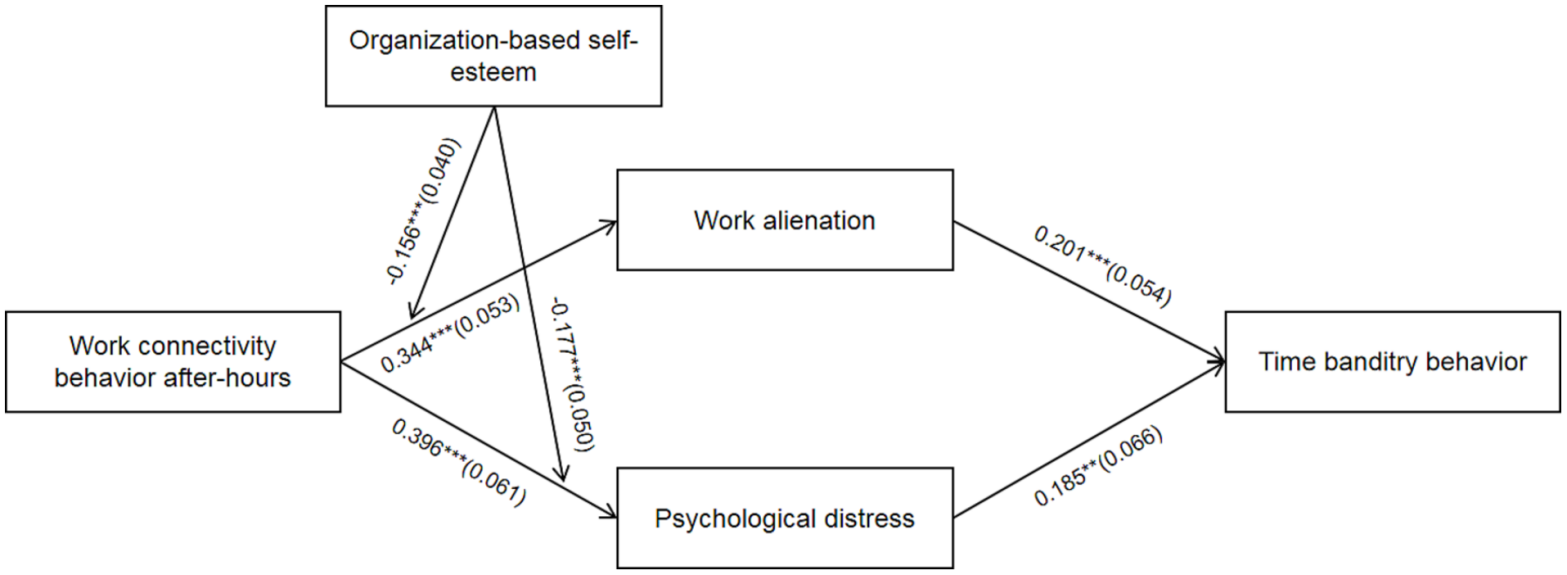
Path coefficients. ^*^p < 0.05; ^**^p < 0 0.01; ^***^p < 0.001; coefficients in the graph are standardized coefficients with standard errors in parentheses; control variables are age, gender and education background.

In this study, Mplus 7.4 was used to test the fitting indexes and related hypotheses of the structural equation model. First, according to the fitting indexes of the theoretical model (*χ*^2^ = 319.988, *df* = 128, *χ*^2^*/df* = 2.500, CFI = 0.944, TLI = 0.933, RMSEA = 0.059, SRMR = 0.060), it can be judged that the fitting of the model is good. Second, the results of the path analysis are shown in Figure 2, work connectivity behavior after-hours is positively correlated with work alienation (*β* = 0.344, *p* < 0.01) and psychological distress (*β* = 0.396, *p* < 0.01), so *H1*, *H2* are verified.

### Mediating effects testing

In this study, Bootstrap (repeated sampling 5,000 times) was used to test the mediating effect of work alienation and psychological distress respectively, and the results are shown in Tables 3, 4. The mediating effect of work alienation (*β* = 0.075, *p* < 0.01) is significant and the 95% confidence interval is [0.034, 0.131], which does not contain 0, so the *H3* is verified. The mediating effect of psychological distress (*β* = 0.078, *p* < 0.05) is significant and the 95% confidence interval is [0.024, 0.164], which does not contain 0. Therefore, *H4* is verified.

**Table 3:** The mediating effect of work alienation.

Model path	*β*	S.E.	*p*	95% Confidence interval	
				Lower	Upper
Total effect	0.195	0.055	0.000	0.093	0.311
Direct effect	0.120	0.051	0.019	0.026	0.233
Indirect effect(WCBA→WA → TBB)	0.075	0.024	0.002	0.034	0.131

*N* = 429; WCBA, work connectivity behavior after-hours; WA, work alienation; TBB, time banditry behavior; Bootstrap = 5,000 times.

**Table 4:** The mediating effect of psychological distress.

Model path	*β*	S.E.	*P*	95% Confidence interval	
				Lower	Upper
Total effect	0.195	0.055	0.000	0.093	0.311
Direct effect	0.117	0.049	0.017	0.026	0.219
Indirect effect(WCBA→PD → TBB)	0.078	0.036	0.030	0.024	0.164

*N* = 429; WCBA, work connectivity behavior after-hours; PD, psychological distress; TBB, time banditry behavior; Bootstrap = 5,000times.

### Moderating effect test

**Figure 3:**
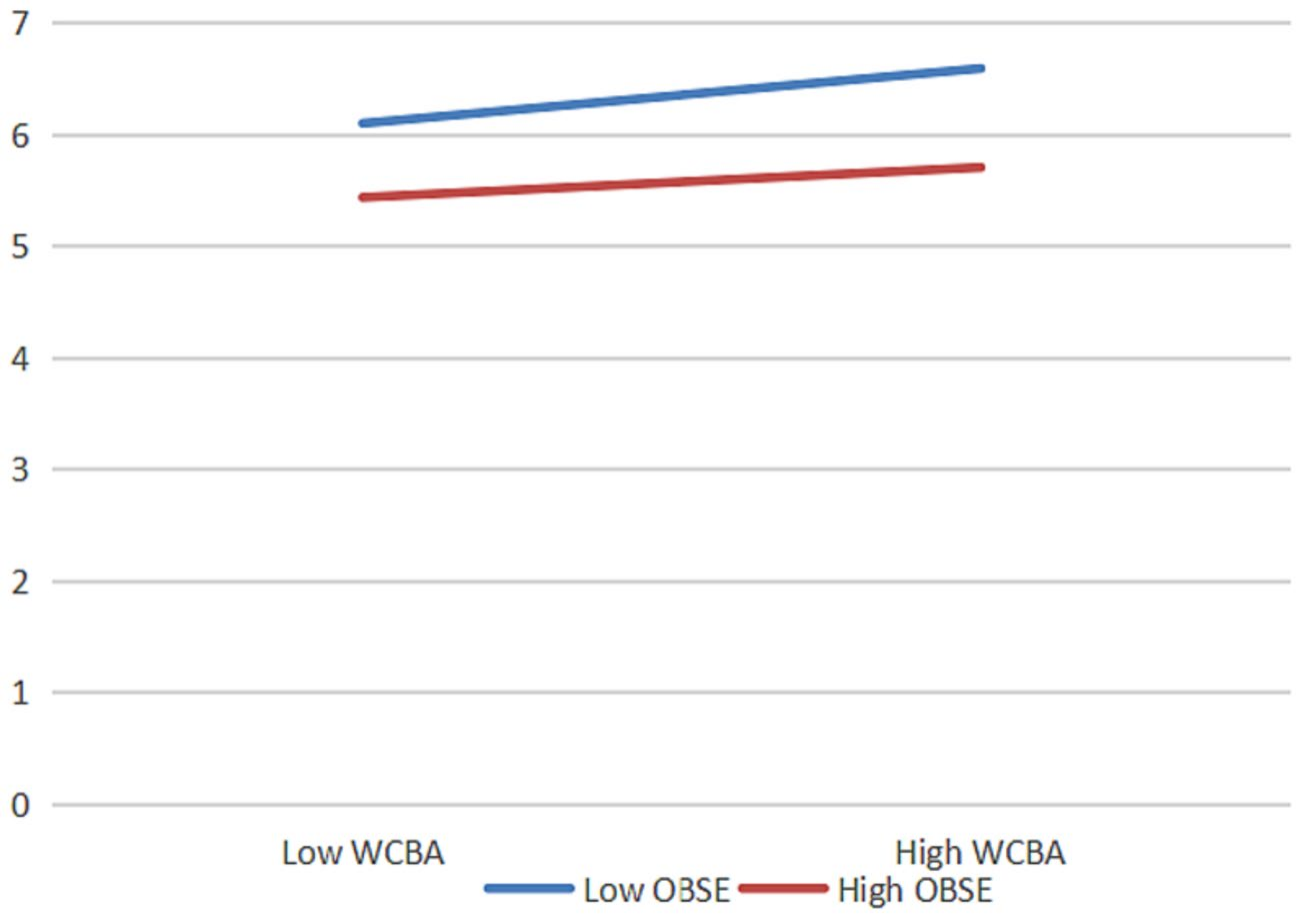
The moderating effect of organizational self-esteem on work connectivity behavior after-hours and work alienation. *N* = 429; WCBA, work connectivity behavior after-hours; OBSE, organization-based self-esteem.

**Figure 4:**
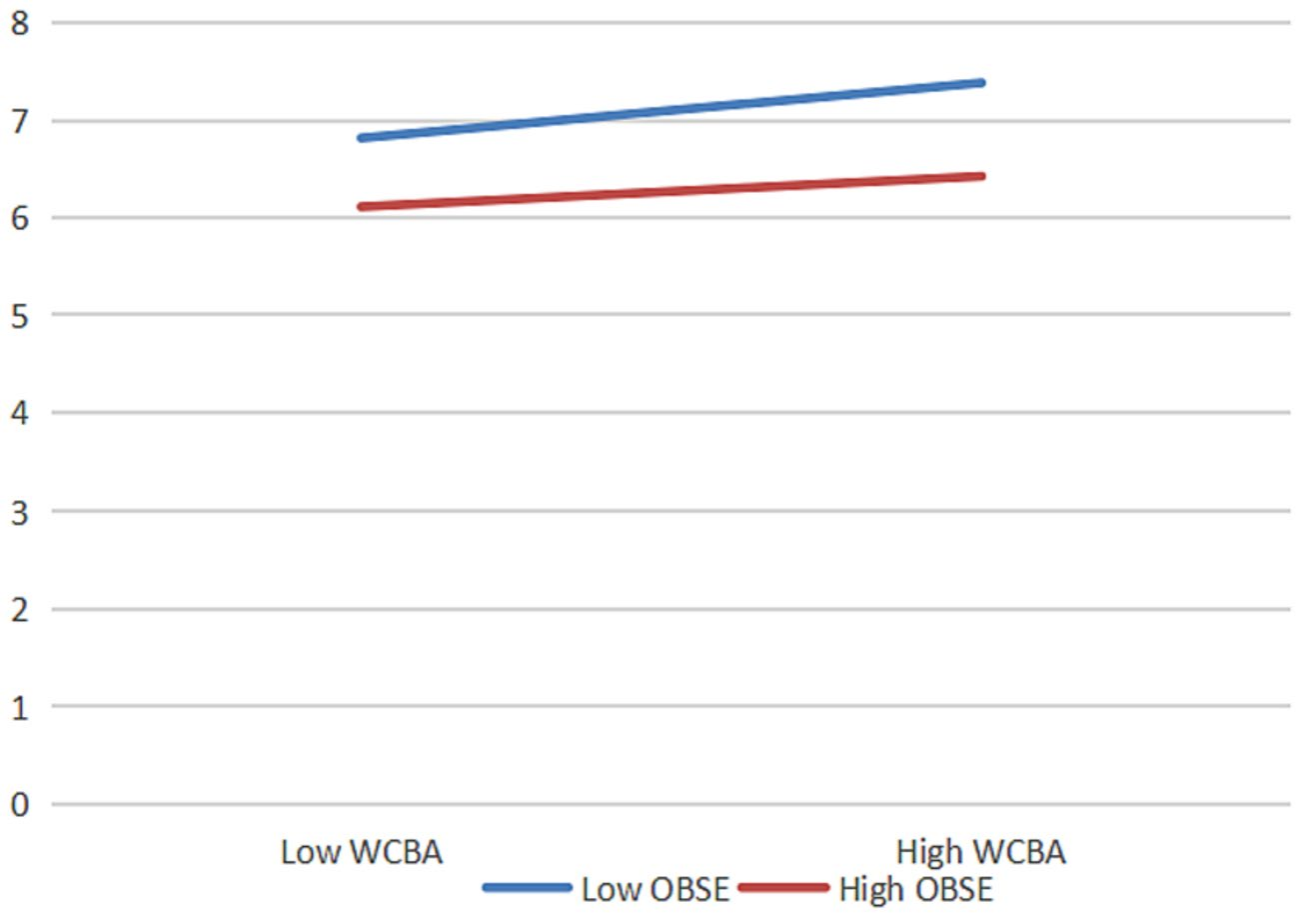
The moderating effect of organizational self-esteem on work connectivity behavior after-hours and psychological distress. *N* = 429; WCBA, work connectivity behavior after-hours; OBSE, organization-based self-esteem.

From Figure 2, it can be concluded that the interaction term between work connectivity behavior after-hours and organization-based self-esteem has a significant path effect on work alienation (*β* = −0.156, *p* < 0.001) and psychological distress (*β* = −0.177, *p* < 0.001), indicating that organization-based self-esteem significantly moderates the relationship between work connectivity behavior after-hours with work alienation and psychological distress. To further explain the moderating effect relationship of organization-based self-esteem, a simple slope test was conducted as suggested by Aiken and West (1991) and plotted as shown in Figures 3, 4.

The results show that when organization-based self-esteem is low, the positive effects of work connectivity behavior after-hours on work alienation (*β* = 0.552, *t* = 8.936, *p* < 0.001) and psychological distress (*β* = 0.632, *t* = 10.161, *p* < 0.001) are stronger. When organization-based self-esteem is high, the positive effects of work connectivity behavior after-hours on work alienation (*β* = 0.137, *t* = 1.910, *p* = 0.057) and psychological distress (*β* = 0.160, *t =* 2.221, *p <* 0.05) are insignificant or weaker. That is, the higher the employees’ organization-based self-esteem is, the weaker the positive effects of work connectivity behavior after-hours on work alienation and psychological distress are, so *H5* and *H6* are verified.

To test the moderating effect of organization-based self-esteem, this study used bootstrap (repeated sampling 5,000 times) to test the moderated mediating effect. The results are shown in Tables 5, 6, where the mediating effect of work alienation between work connectivity behavior after-hours and employees’ time banditry behavior is moderated by organization-based self-esteem, i.e., for employees with high organization-based self-esteem (one standard deviation above the mean), the indirect effect of work connectivity behavior after-hours via work alienation on their time banditry behavior is significantly lower than for employees with low organization-based self-esteem (one standard deviation below the mean), and the difference is significant (*β* = −0.083, *p* < 0.05) and the 95% confidence interval is [−0.164, −0.033], excluding 0. Thus, *H7* is tested.

**Table 5:** Test results for moderated chain mediated effects (a).

Moderator variable	Path: WCBA→WA → TBB				
	*β*	S.E.	*p*	95% Confidence interval	
				Lower	Upper
Low OBSE	0.111	0.035	0.001	0.051	0.187
High OBSE	0.027	0.019	0.141	−0.003	0.070
Differences	−0.083	0.033	0.012	−0.164	−0.033

*N* = 429; WCBA, work connectivity behavior after-hours; WA, work alienation; TBB, time banditry behavior; OBSE, organization-based self-esteem; Bootstrap = 5,000 times.

**Table 6:** Test results for moderated chain mediated effects (b).

Moderator Variable	Path: WCBA→PD → TBB				
	*β*	S.E.	*p*	95% Confidence interval	
				Lower	Upper
Low OBSE	0.117	0.052	0.023	0.037	0.240
High OBSE	0.030	0.019	0.128	0.000	0.077
Differences	−0.087	0.044	0.045	−0.200	−0.025

*N* = 429; WCBA, work connectivity behavior after-hours; PD, psychological distress; TBB, time banditry behavior; OBSE, organization-based self-esteem; Bootstrap = 5,000 times.

The mediating effect of psychological distress between work connectivity behavior after-hours and employees’ time banditry behavior is moderated by organization-based self-esteem, i.e., for employees with higher organization-based self-esteem (one standard deviation higher than the mean), the indirect effect of work connectivity behavior after-hours via psychological distress on employees’ time banditry behavior is significantly lower than for employees with lower organization-based self-esteem (one standard deviation lower than the mean), and the difference is significant (*β* = −0.087, *p <* 0.05) and the 95% confidence interval is [−0.200, −0.025], excluding 0. Therefore, *H8* is verified.

## Discussion

### Theoretical implications

First, this study expands the outcome variables and research perspectives of work connectivity behavior after-hours. Although previous studies have explored the impact outcomes of work connectivity behavior after-hours, they have mainly focused on employees’ health (Dettmers et al., 2016), family (Derks et al., 2016), and emotions (Dettmers et al., 2016), and there is a relative lack of research on the impact of employee behavioral dimensions, and few scholars have confirmed the impact of work connectivity behavior after-hours on employees’ negative extra-role behaviors, such as employees’ time banditry behaviors. With the normalization of work connectivity behavior after-hours, empirical studies conducted in this area are relatively insufficient, while this study found the positive effect of work connectivity behavior after-hours on employees’ time banditry behavior through empirical analysis, which to some extent expands the research on the effect of work connectivity behavior after-hours.

Second, this study strengthens the explanatory power of Conservation of Resources Theory in the field of work connectivity behavior after-hours research. Previous studies have indicated that high work demands lead to stress and are highly positively associated with work alienation and psychological distress (Duffy et al., 2002; Gozukara et al., 2017), and the present study confirms this view that when work connectivity behavior after-hours is a job requirement, it directly or indirectly depletes employees’ limited resources and triggers work alienation and psychological distress. The resulting sense of resource deprivation and imbalance prompts employees to seek new resources and engage in employees’ time banditry behaviors such as taking extra breaks or attending to personal matters during work hours, revealing the specific mechanisms by which work connectivity behavior after-hours on employees’ time banditry behaviors. In addition, the current research on the factors influencing employees’ time banditry behaviors mostly focuses on interpersonal stressors and negative leadership styles (Ding et al., 2018), etc. This study explores the mechanisms of the influence of work-level factors on employees’ time banditry behaviors from work connectivity behavior after-hours and further expands the study of antecedent variables of employees’ time banditry behaviors.

Finally, this study explores the moderating effect of organizational-based self-esteem. At present, research on organizational-based self-esteem is more focused on exploring independent or mediating variables (Jahanzeb and Bouckenooghe, 2023; Sujatha et al., 2023), and this study finds that organizational-based self-esteem increases employees’ psychological resource reserves when it is used as a personal resource, and to some extent, it replenishes some of the emotional resources depleted by work connectivity behavior after-hours and weakens the positive effects of work connectivity behavior after-hours on work alienation and psychological distress as well as the mediating effects of work alienation and psychological distress between work connectivity behavior after-hours and employees’ time banditry behaviors, respectively. This study enriches the research on organization-based self-esteem to a certain extent, and also provides new solutions for organizations to weaken the negative effects of work connectivity behavior after-hours on work alienation and psychological distress.

### Practical implications

First, the organization should explores a scientific and reasonable work system and a matching compensation system. On the one hand, the organization can give employees the right to refuse to deal with work-related connectivity during non-working hours through explicit provisions without coercion or pressure, giving employees full autonomy and reducing the tension and boredom of employees forced to respond (Sonnentag and Niessen, 2020). On the other hand, the organization should limit the frequency of electronic communication between departments during non-working hours to maintain employees’ rest rights. In addition, in order to reduce employees’ perception of unfairness, the organization can also count the workload received and completed by employees during non-working time communication as part of their normal workload and give them certain compensation to prevent them from time banditry due to psychological imbalance. In addition to reducing employees’ sense of resource loss, the organization should also provide employees with additional resources through various channels to help individuals recover their resources. For example, an effective communication and feedback mechanism should be established so that employees’ emotional changes can be noticed in a more timely manner, and employees with negative emotions due to excessive work stress can be communicated and guided in a timely manner, and leave can be arranged for them. For employees, they need to make efficient use of work time to achieve their work goals, so as to reduce the frequency of work connectivity behavior after-hours and avoid falling into the vicious circle of working overtime during the non-working time to deal with work and spending time at work.

Second, the organization should pay attention to the working growth environment, and pay attention to the physical and mental health development of employees. On the one hand, the organization should strive to enhance the sense of ownership of employees, so that employees firmly explores a “unit is my home, development depends on everyone” concept. The organization should build a harmonious interpersonal relationship through various activities, enhance mutual trust and collaboration among members, and reduce employees’ sense of personal and social alienation (He and Zhou, 2023). On the other hand, the organization should set up psychological mediator positions according to the actual situation, specifically responsible for the psychological counseling of employees, to assist employees to get rid of emotional distress and avoid falling into the vicious circle brought about by work connectivity behavior after-hours. In addition, it can also enrich its own work by increasing the content of employees’ work and setting diverse goals, so as to stimulate and mobilize employees’ enthusiasm and motivation (Sun et al., 2023).

Third, the organization should try to avoid a series of behaviors brought about by employees’ negative emotions that are detrimental to the interests of the organization or other organizational members. On the one hand, the organization should reasonably guide employees to seek the meaning of work, help them vent their negative emotions and avoid their possible negative extra-role behaviors; on the other hand, the organization should also pay attention to creating a positive and time-valuing work atmosphere. Once the phenomenon of “dilly-dallying” becomes the norm, it will definitely bring irreparable loss to the organization. In view of this, organizations should be set clear rules and regulations and corresponding reward and punishment mechanisms to strengthen employees’ awareness of time management and reduce the tolerance of time encroachment within the organization.

Fourth, organizations should regularly monitor employees’ organization-based self-esteem levels and develop individualized management policies. On the hand, for employees with lower levels, managers should respect their subject status and value, encourage them to actively participate in decision making by implementing positive motivation, not shying away from praise, and showing more importance to them, so that employees recognize themselves as meaningful and important employees to the organization and promote them to develop higher organization-based self-esteem and motivate them to work. On the other hand, for employees with higher levels of organization-based self-esteem, give them some space for work autonomy and other resources to provide opportunities and guarantees to promote organization-based performance improvement (Jahanzeb and Bouckenooghe, 2023).

### Limitations and directions for future research

There are some limitations and shortcomings in this study that need to be continued and improved in subsequent studies. Firstly, this study confirms the relationship between work connectivity behavior after-hours research and employees’ time banditry behavior. In the future, other perspectives can be further introduced to explore variables that have not yet been addressed, such as work family conflict, stress, etc., (Pascucci et al., 2021).

Secondly, this study adopts a cross-sectional research design, which may not accurately reveal the dynamic processes among variables, and the persuasiveness of the findings can be improved in the future through methods such as the experience sampling method and logbook method.

Thirdly, the respondents of this study come from various industries, and the ways of work connectivity behavior after-hours and employees’ time banditry behavior may differ among employees in different industries and different types of employees (e.g., knowledge workers and manual workers). Therefore, the findings of this study can be further validated in the future for employees in a particular industry or for a particular type of employees.

Fourthly, the importance of content factors of work connectivity behavior after-hours, such as verbal factors (e.g., formal or informal greetings) and non-verbal factors (use of emojis), was ignored in the measurement, and the impact of content-based work connectivity behavior after-hours on employees’ attitudes and behaviors could be examined in the future.

Finally, the data in this study were obtained from employees in Chinese companies, and further cross-cultural research is needed to determine the applicability of the findings in other cultural contexts.

## Conclusion

Many previous studies have emphasized the impact of non working time connected behavior on employee role within behavior (Pauli et al., 2023), but we still know very little about the causal relationships that affect employee role outside behavior. The purpose of this study was to explore the mechanisms and boundary conditions of the influence of work connectivity behavior after-hours on employees’ time banditry behaviors from the perspective of Conservation of Resources Theory. The survey data support the mediating role of work alienation and psychological distress between work connectivity behavior after-hours and employees’ time banditry behaviors and the moderating role of organizational self-esteem, respectively. Work connectivity behavior after-hours consumes employee individual resources, triggers feelings of work alienation and psychological distress, and leads to employees’ time banditry behaviors during working hours, and in this process, organizational self-esteem effectively weakens the positive effects of work connectivity behavior after-hours on feelings of work alienation and psychological distress and mediates the effects of work alienation and psychological distress.

## Data availability statement

The original contributions presented in the study are included in the article/supplementary material, further inquiries can be directed to the corresponding author.

## Ethics statement

Ethical review and approval was not required for the study on human participants in accordance with the local legislation and institutional requirements. Written informed consent from the patients/ participants or patients/participants legal guardian/next of kin was not required to participate in this study in accordance with the national legislation and the institutional requirements.

## Author contributions

HC: Data curation, Investigation, Project administration, Visualization, Writing-original draft, Writing-review & editing. LW: Investigation, Resources, Writing-review & editing. JB: Formal analysis, Investigation, Methodology, Resources, Writing-review & editing. JL: Methodology, Validation, Writing-review & editing.
